# The relationship between preoperative T helper cytokines in the ileal mucosa and the pathogenesis of pouchitis

**DOI:** 10.1186/s12876-020-01421-w

**Published:** 2020-08-18

**Authors:** Takahito Kitajima, Yoshiki Okita, Mikio Kawamura, Satoru Kondo, Yuji Toiyama, Keiichi Uchida, Masato Kusunoki

**Affiliations:** Department of Gastrointestinal and Pediatric Surgery, Division of Reparative Medicine, Institute of Life Sciences, Mie University Graduate School of Medicine, 2-174 Edobashi, Tsu, Mie 514-8507 Japan

**Keywords:** Ulcerative colitis, Pouchitis, Cytokine, IFN-γ, IBD

## Abstract

**Background:**

Although the etiology of pouchitis remains unknown, inflammatory cytokines are significantly associated with the pathogenesis of pouchitis. The cytokine responses that characterize inflammatory bowel diseases (IBD) are key pathogenic components of the disease. Although cytokine profiles in the colonic mucosa have been investigated in experimental colitis models or IBD patients, cytokine profiles in the ileal mucosa at colectomy have been rarely assessed.

**Aim:**

To assess the relationship between pouchitis and T helper (Th) cytokines in the ileal mucosa collected at the time of colectomy and pouch construction.

**Methods:**

This retrospective study involved 68 consecutive patients from January 2004 to May 2011 who underwent ileal pouch–anal anastomosis for ulcerative colitis. Samples were obtained from the terminal ileum of resected specimens at time of total colectomy or subtotal colectomy. mRNA expression levels of Th cytokines (IFN-γ, IL-23A, IL-5, IL-13 and IL-17A) were determined.

**Results:**

Forty of 68 patients (58.8%) developed pouchitis. There was no association between IL-23A expression levels and incidence of pouchitis (*p =* 0.301). Patients with elevated IFN-γ had a significantly higher incidence of pouchitis compared with low IFN-γ patients (*p* = 0.043). Univariate analysis demonstrated a total dose of prednisolone > 7000 mg administered before colectomy (*p* = 0.04) and high IFN-γ expression (*p* = 0.02) were significant risk factors for pouchitis onset. In multivariate analysis, elevated IFN-γ messenger(m)RNA levels were significantly associated with pouchitis onset (*p* = 0.03).

**Conclusion:**

IFN-γ expression in the normal ileal mucosa at the time of colectomy may be an important factor in the pathophysiology of pouchitis.

## Background

Restorative proctocolectomy and ileal pouch anal anastomosis (IPAA) have been established as standard surgical treatment methods for the management of ulcerative colitis (UC). Pouchitis, a nonspecific inflammatory condition of the ileal pouch, is a well-known complication in patients who undergo IPAA. Pouchitis is defined as nonspecific inflammation developing after intestinal continuity of IPAA. The incidence of pouchitis is 40–70% in UC patients who receive IPAA [1–3]. Although the immunopathogenesis of pouchitis is unclear, there are two major mechanisms associated with pouchitis: microbial dysbiosis and abnormal immune responses [4]. Whether inflammation of the pouch mucosa represents a recurrence of immune mechanisms in UC or a new form of inflammatory bowel disease (IBD) remains a topic of discussion.

Cytokine responses that characterize IBD are key pathogenic components of the disease. Cytokine responses play key roles in the initiation, evolution, maintenance, and resolution of inflammation. Classically, two types of cytokine responses, determined by T-cell differentiation, have been described [5]: T-helper cell (Th)1 and Th2. Th1, characterized by the production of interferon (IFN)-γ mediates Crohn’s disease (CD) [6]. Th2 responses, characterized by the production of interleukin (IL)-5 and IL-13 as well as normal IFN-γ, mediate UC [7, 8]. In addition to these two Th cell types, the recently described Th17 cells, producing IL-17 and IL-23, were reported as a third component of Th cells in various autoimmune disease models [9–11]. IL-23 is a newly discovered cytokine that has a role in the maintenance and/or expansion of Th17 cells [12, 13]. In the mucosa of IBD, Th17 cells have been proposed to play a key role in intestinal inflammation [14, 15]. Th1 cytokines as well as those secreted from Th17 cells might be associated with CD [14]. Olsen et al. reported that the gene expression levels of IL-17A, IL-23 and IFN-γ correlated with the grade of inflammation in UC, and that IL-17A and IL-23 had a role in mediating inflammation in both forms of IBD [16]. Clarification of these complex networks of Th cytokines might lead to the identification of novel targets for the diagnosis and treatment of CD and UC.

Mucosal concentrations of proinflammatory cytokines including IL-1ß, IL-6 and IL-8 were increased in pouchitis similar to UC [17, 18]. However, cytokine profiles in the ileal mucosa at the time of colectomy have been rarely assessed. Investigating these cytokines in the ileal mucosa might help diagnose the subsequent onset of pouchitis. To the best of our knowledge, no study has examined Th cytokine profiles in ileal mucosa obtained at the time of total colectomy in patients with UC. This study evaluated Th cytokines in the ileal mucosa from UC patients at the time of total colectomy, and tested the predictive value of these cytokines for the onset of pouchitis.

## Methods

### Patients and specimens

From 2004 to 2011, this study included 183 patients who underwent IPAA in our institution. One hundred fifteen patients excluded for the following reasons: unable to obtain the frozen tissue samples of ileal mucosa because of emergency surgery (subtotal colectomy) (*n* = 65), poor quality of samples(*n* = 12) and lost to follow-up(*n* = 38). In total, frozen tissue samples of ileal mucosa were obtained from 68 patients who underwent IPAA in our institution. Samples were obtained from the terminal ileum of resected specimens at time of total colectomy or subtotal colectomy. Patients with obvious backwash ileitis were excluded. Samples were soaked in RNA later and stored at − 80 °C until RNA extraction. The protocol for this research project has been approved by a suitably constituted Ethics Committee of the institution and it conforms to the provisions of the Declaration of Helsinki. All informed consent was obtained from the subjects and guardians.

### Surgical procedure and diagnosis of pouchitis

All patients underwent IPAA and routinely performed endoscopy each year after stoma closure. In addition, patients with suspected pouchitis were assessed by endoscopic assessments. Patients with pouchitis were diagnosed by a modified Pouchitis Disease Activity Index (mPDAI) score ≥ 5 [19]. Patients with CD of pouch were diagnosed with granulomas, stenosing, fistulising and inflammatory during follow-up [20, 21] . Patients with CD of the pouch and Secondary pouchitis (pouch ischemia, anastomotic stricture, pelvic sepsis, *Cytomegalovirus* infection, *Clostridium difficile* infection, and regular use of nonsteroidal anti-inflammatory drugs) were excluded. The onset of pouchitis was defined as the time from stoma closure to clinical and endoscopic diagnosis at the first episode. Acute antibiotic-responsive pouchitis (ADP) was defined as episodes of symptomatic pouch inflammation occurring fewer than 4 times per year with symptomatic response to a 2-week course of a single antibiotic such as ciprofloxacin. Chronic antibiotic-refractory pouchitis (CARP) included patients with greater than 4 episodes of symptomatic pouch inflammation per year, individuals who require continuous antibiotic therapy to maintain symptom remission, or patients whose symptoms were refractory to antibiotic therapy [22].

### RNA extraction and cDNA synthesis

Ileal mucosa was homogenized with a Mixer Mill MM 300 homogenizer (Qiagen, Chatsworth, CA, USA). Total RNA was isolated using a RNeasy Mini Kit (Qiagen) according to the manufacturer’s instructions. Then, purity and concentration of RNA was estimated with Nano-Drop. Pure RNA was obtained with a A260/A280 ratio of 2.0–2.2. Degraded RNA could be detected with different ratio. cDNA was synthesized from 5.0 mg total RNA with random hexamer primers and Superscript III reverse transcriptase (Invitrogen, Carlsbad, CA) according to the manufacturer’s instructions.

### Quantitative reverse transcription-polymerase chain reaction (qRT-PCR)

qRT-PCR analysis was performed using the TaqMan universal PCR Master Mix (Applied Biosystems, Foster City, CA, USA). We investigated the mRNA expression of 5 cytokines in the terminal ileum focusing on Th2 responses (IL-5, IL-6, IL-13, IL-17A and IL-23), and IFN-γ. The relative abundance of target transcripts was measured using TaqMan probes (Applied Biosystems, Foster City, CA, USA) for IFN-γ (Assay ID: Hs00989291_m1), IL-5 (Assay ID: Hs01548712_g1), IL-13 (Assay ID: Hs00174379_m1), IL-17A (Assay ID: Hs001743883_m1), and IL-23A (Assay ID: Hs00900828_g1). Glyceraldehyde-3-phosphate dehydrogenase: *GAPDH* (Assay ID, Hs02758991_g1; Applied Biosystems) was measured as an internal housekeeping gene. cDNA was amplified and quantified using Applied Biosystems StepOne Plus Real-Time PCR System and analyzed by Software version 2.2.2 (Applied Biosystems).

### Quantification of the relative expression levels of Th cytokines

Relative gene expression was determined using the standard curve method. Standard curves and line equations were generated using five-fold serially diluted solutions of cDNA generated by the reverse transcription of qPCR Human Reference Total RNA (Clontech, Mountain View, CA, USA). All standard curves were linear in the analyzed range with an acceptable correlation coefficient (*R*^2^). Target gene expression was calculated from the standard curve followed by the quantitative normalization of cDNA in each sample using *GAPDH* as an internal control. Assays were performed in duplicate for each sample and the mean value was used for analysis.

### Statistical analysis

Results were expressed as median values (interquartile range). The cutoff value of each continuous variable was determined by the median value. Comparisons were performed using non-parametric Wilcoxon signed-rank test for continuous variables. Cumulative incidence of pouchitis was evaluated by the Kaplan-Meier method. Differences between two groups were determined by the log-rank test. Cox proportional hazard regression analysis was used to evaluate the independent influence of factors on the onset of pouchitis. All statistical analyses were carried out using JMP 10 for Windows software (SAS Institute, Cary, NC, USA). Two-sided *p*-values < 0.05 were considered statistically significant.

## Results

### Patient characteristics

**Fig. 1:**
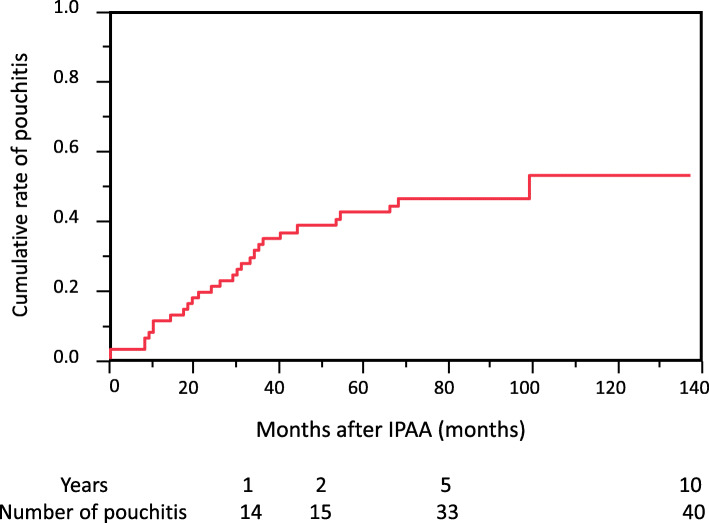
Cumulative risk of developing pouchitis. Cumulative risk of developing pouchitis was 58.8% at the follow-up period (median 40 months (0–117 months))

Sixty-eight patients (male/female; 39/29, median age; 32 years (11–62 years)) were enrolled in this study. The median follow-up period was 40 months (0–117 months). Among these patients, 40 (58.8%) developed pouchitis (Fig. 1). Patient characteristics are shown in Table 1.

**Table 1:** Clinicopathological factors of 68 patients with pouchitis

Features	pouchitis (*n* = 40)	Non-pouchitis (*n* = 28)	*P*-value
Age at IPAA (years)	35.25 ± 2.12	33.00 ± 2.34	0.562
Gender (Male/female)	23/17	16/12	0.976
Disease duration (years)	8.54 ± 1.35	9.57 ± 1.83	0.635
Extent of colitis (total colitis/left-sided colitis, proctitis)	30/10	24/4	0.282
Severity of colitis (severe/moderate or mild)	6/34	3/25	0.607
Extra intestinal manifestation (yes/no)	1/39	1/27	0.066
Total dasage of steroid before IPAA (g)	15.66 ± 3.41	18.39 ± 3.16	0.268
Dosage of steroid per month just before surgery (mg)	486.3 ± 91.9	449.8 ± 83.8	0.659
Immunomodulater use (yes/no)	20/27	21/16	0.195
White blood cells (× 10^3^/μl)	8.53 ± 0.77	8.96 ± 0.62	0.190
Hemoglobin (g/dl)	10.9 ± 0.41	10.8 ± 0.33	0.717
Platelets (×10^4^/μl)	31.4 ± 1.62	35.4 ± 1.97	0.068
Albumin (g/dl)	3.50 ± 0.11	3.44 ± 0.11	0.644
C-reactive proten (mg/dl)	2.16 ± 0.75	0.78 ± 0.16	0.426
Anastomotic leakage (yes/ no)	5/35	2/26	0.474

Categorical data were compared by chi-square test or Fisher’s exact test

Bold text indicates statistical significance, *p < 0.05

Abbreviations; *IPAA* Ileal pouch anal anastomosis

### Expression levels of Th cytokines

The mRNA expression levels of Th cytokines (IFN-γ, IL-23A, IL-5, IL-13 and IL-17A) were calculated. More than half of the samples were below the level of detection in IL-5, IL-13 and IL-17A assays. These cytokines were excluded from further analysis.

### Cumulative incidence of pouchitis after IPAA according to mRNA levels of Th cytokines

**Fig. 2:**
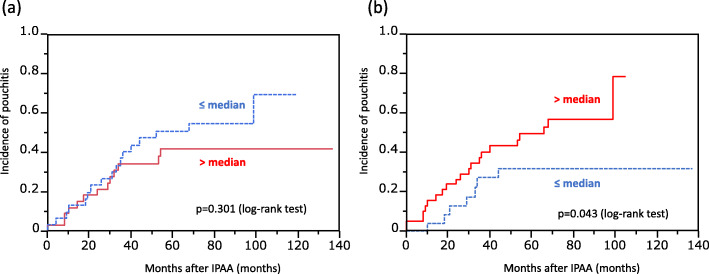
The relationship between the cumulative incidence of pouchitis and IL-23A (**a**) and IFN-γ (**b**). There was no association between IL-23A expression levels and the incidence of pouchitis (*p =* 0.301) (a). However, the patients with elevated IFN-γ had a significantly higher incidence of pouchitis than those with low levels of IFN-γ (*p* = 0.043)

The cumulative incidence of pouchitis according to the expression levels of IFN-γ and IL-23A are shown in Fig. 2. Although there was no association between IL-23A expression levels and the incidence of pouchitis (*p =* 0.301), patients with elevated IFN-γ had a significantly higher incidence of pouchitis than those with low levels of IFN-γ (*p* = 0.043). Because only IFN-γ was associated with the onset of pouchitis, we focused the rest of our study on IFN-γ.

### Relationship between IFN-γ and preoperative clinicopathologic data

The relationship between pouchitis and preoperative clinicopathologic factors is shown in Table 2. There was a significant difference between IFN-γ level and severity of colitis, and patients with elevated C-reactive protein had a trend for higher IFN-γ mRNA levels. However, there was no correlation between IFN-γ and type of pouchitis. According to univariate analysis, a total dose of prednisolone > 7000 mg administered before colectomy and IFN-γ were identified as significant risk factors for the onset of pouchitis (Table 3). In addition, multivariate analysis showed that elevated IFN-γ mRNA levels were significantly associated with the onset of pouchitis.

**Table 2:** Relationship between IFN^†^-γ mRNA expression and preoperative clinicopathologic factors

variable		n	mRNA expresion (mean ± SD)	*P*-value
Gender	Male	39	0.99 ± 0.13	0.98
	Female	29	1.19 ± 0.22	
Age (years)	> 32 (median)	34	0.97 ± 0.14	0.77
	≤32	34	1.16 ± 0.19	
Disease duration	> 7 years (median)	33	0.91 ± 0.16	0.25
	≤7 years	35	1.17 ± 0.18	
Extent of colitis	Total colitis	54	1.13 ± 0.14	0.16
	Left side colitis, proctitis	14	0.64 ± 0.08	
Severity of colitis	Severe	9	1.88 ± 0.52	0.04*
	Moderate, mild	59	0.90 ± 0.10	
Matts’ grade	3,4	38	1.19 ± 0.23	0.18
	1,2	28	0.85 ± 0.12	
Extra intestinal manifestation	Yes	2	1.17 ± 0.36	0.38
	No	66	1.02 ± 0.12	
Total dasage of steroid before surgery (mg)	> 7000	39	0.95 ± 0.13	0.98
	≤7000	29	1.14 ± 0.22	
Immunomodulator use	Yes	27	1.00 ± 0.19	0.90
	No	41	1.04 ± 0.16	
White blood cells(/μl)	> 9000	27	1.11 ± 0.20	0.45
	≤9000	41	0.97 ± 0.15	
CRP^‡^ (mg/dl)	≥0.5	32	1.11 ± 0.16	0.07
	< 0.5	35	0.83 ± 0.13	
Type of pouchitis	No pouchitis	38	1.04 ± 0.40	0.36
	Acute antibiotic-responsive pouchitis	17	1.14 ± 0.50	
	Chronic antibiotic-refractory pouchitis	13	0.90 ± 0.30	

Categorical data were compared by chi-square test or Fisher’s exact test

Bold text indicates statistical significance, *p < 0.05

Abbreviations: ^†^*IFN* Interferon; ^‡^*CRP* C-reactive protein

**Table 3:** Univariate and multivariate analysis of factors influencing pouchitis

Factors	Univariate			Multivariate		
	Odd ratio	95%CI	*p* Value	Odd ratio	95%CI	*p* Value
Gender (male vs female)	1.21	0.46–3.24	0.70			
Age (≤32 years vs < 32 years)	0.48	0.17–1.27	0.14			
Disease duration (≤7 years vs < 7 years)	0.68	0.25–1.80	0.44			
Extent of colitis (total colitis vs Left side colitis, proctitis)	2.32	0.68–9.30	0.18			
Severity of colitis (severe vs moderate, mild)	1.68	0.40–8.59	0.48			
Matts’ grade (3,4 vs 1,2)	0.92	0.34–2.48	0.88			
Total dosage of steroid before surgery (≤7000 mg vs < 7000 mg)	2.59	1.01–7.34	0.04*	2.57	0.92–7.57	0.06
Immunomodulator use (Yes vs No)	2.16	0.81–5.94	0.12			
IL-23A mRNA (≤median vs < median)	0.55	0.68–1.45	0.23			
IFN-γ mRNA (≤median vs < median)	3.05	1.11–8.92	0.02*	3.04	1.08–9.12	0.03*

*CI* Confidence interval; mod. and well, moderately and well differentiated

Bold text indicates statistical significance, *p < 0.05

## Discussion

The etiology of pouchitis is unknown, but numerous risk factors have been identified. Increased incidence of pouchitis in patients with extraintestinal manifestations [23–27], younger age [27], shorter disease duration [2], extent of disease [2, 27], more severe preoperative clinical course, steroid dependency [28] and primary sclerosing cholangitis [29–31] have been reported. Other studies have reported that even pre- and postoperative use of biologics [32], steroid use before colectomy [26, 27], and postoperative use of nonsteroidal anti-inflammatory drugs [25], have significantly correlated with pouchitis. However, in our study, these factors are no statistical difference between the pouchitis and no pouchitis groups. Therefore, additional risk factors or aetiologic factors are needed to help predict which patients are at risk of pouchitis. In our study, only IFN-γ mRNA expressions in patients who developed pouchitis were significantly higher than in patients without pouchitis (*p* = 0.043). In addition, multivariate analysis demonstrated that elevated IFN-γ mRNA levels was an independent risk factor for the onset of pouchitis.

Pouchitis is a heterogeneous disorder with a combination of various underlying case. Acute pouchitis may be more attributable to dysbiosis in the ileal pouch. ADP, which is predominately microbially mediated, requires antibiotic therapy to maintain symptom remission. On the other hands, CARP may be a result of abnormal host immune response. Thus, a subgroup of patients with CARP does not respond to standard antibiotic therapy [4, 33–35]. The treatment of choice is immunosuppressive therapy with a good response in the treatment of CARP and Crohn’s pouchitis [36–38]. Therefore, when discussing risk factors or predicting factors for pouchitis, it is important to accurately phenotype patients with pouchitis. Our data suggest that IFN-γ expression in ileal mucosa is slightly lower in patients with CARP than those with ADP, although not significant, since Crohn’s pouchitis excluded in this study. Further large number study are needs to confirm the accurate correlation between IFN-γ and immune mediated pouchitis.

IFN-γ, a cytokine secreted by activated T cells and natural killer (NK) cells, promotes inflammation by activating macrophages and upregulating the expression of cell adhesion molecules [39]. Furthermore, increased IFN-γ production might be responsible for the tissue damage observed in pouchitis because IFN-γ sustains cytotoxic reactions [40]. Signal transducer and activator of transcription (STAT)1 is part of the signaling pathway of other cytokines/growth factor receptors as well as be a hallmark of IFN-γ receptor signal transduction [41–43]. Previous studies investigating activation and expression of nuclear factor-kappa B (NF-κB) and members of the STAT family has positive correlation with the activation of cytokine transcription factors in IBD [41–43]. Although increased NF-κB activation was more predominant in CD than in UC, activation and expression of STAT1 were increased in UC compared with CD and normal controls [43–45]. A study investigating intracellular cytokine data reported that the pathophysiology of small-intestinal inflammation occurring after colectomy in patients with UC might be associated with a Th2 cytokine phenotype [46]. Additionally, there was a significant increase in the number of IFN-γ producing mononuclear cells in patients with UC with pouchitis compared with UC patients without pouchitis [47]. Furthermore, there was a tendency towards increased levels of IFN-γ and STAT1 in patients with UC, even without clinical and endoscopic evidence of pouchitis [48]. Of note, the normal pouch already indicated high levels of STAT1 expression and activation compared with normal preoperative ileum [44].

There are some limitations in this cohort. First, this retrospective study included a limited number of patients with geographical reason from a single cohort. We were unable to detect the genuine number of the patients with pouchitis. Therefore, the finding needs to be validated in a larger prospective cohort. Second, the short follow-up time duration was insufficient to evaluate incidence of pouchitis. Third, we analyzed specific 5 cytokines which might be suggested with incidence of pouchitis in this study. Therefore, further comprehensive analyses of ileal mucosal cytokines related with pouchitis are needed. Nevertheless, our findings suggest that IFN-γ expression in the normal ileal mucosa at the time of colectomy may be an important factor in the pathophysiology of pouchitis. However, further proteomics approach such as ELISA, immunohistochemical analysis or Western blot are needed to confirm the real IFN-γ expression in the ileal mucosa.

## Conclusions

Our data suggest that IFN-γ expression in the normal ileal mucosa at the time of colectomy may be an important factor in the pathophysiology of pouchitis. Further research is required to determine the physiological role of IFN-γ.

## Data Availability

All data generated or analyzed during this study are included in this published article.

## Abbreviations

IBD: Inflammatory bowel diseases
Th: T helper
mRNA: Messenger RNA
IPAA: Ileal pouch anal anastomosis
UC: Ulcerative colitis
IFN: Interferon
CD: Crohn’s disease
IL: Interleukin
ADP: Antibiotic dependent pouchitis
CARP: Chronic antibiotic refractory pouchitis
NF-κB: Nuclear factor-kappa B
STAT: Signal transducer and activator of transcription
